# Material Optimization and Curing Characterization of Cold-Mix Epoxy Asphalt: Towards Asphalt Overlays for Airport Runways

**DOI:** 10.3390/polym17152038

**Published:** 2025-07-26

**Authors:** Chong Zhan, Ruochong Yang, Bingshen Chen, Yulou Fan, Yixuan Liu, Tao Hu, Jun Yang

**Affiliations:** 1 School of Transportation, Southeast University, #2 Southeast University Road, Nanjing 211189, China; 2 Department of Civil and Environmental Engineering, The Hong Kong Polytechnic University, Kowloon, Hong Kong

**Keywords:** airport overlay, cold-mix epoxy asphalt, curing behaviors, non-isothermal curing kinetics, differential scanning calorimetry (DSC)

## Abstract

Currently, numerous conventional airport runways suffer from cracking distresses and cannot meet their structural and functional requirements. To address the urgent demand for rapid and durable maintenance of airport runways, this study investigates the material optimization and curing behavior of cold-mix epoxy asphalt (CEA) for non-disruptive overlays. Eight commercial CEAs were examined through tensile and overlay tests to evaluate their strength, toughness, and reflective cracking resistance. Two high-performing formulations (CEA 1 and CEA 8) were selected for further curing characterization using differential scanning calorimetry (DSC) tests, and the non-isothermal curing kinetics were analyzed with different contents of Component C. The results reveal that CEA 1 and CEA 8 were selected as promising formulations with superior toughness and reflective cracking resistance across a wide temperature range. DSC-based curing kinetic analysis shows that the curing reactions follow an autocatalytic mechanism, and activation energy decreases with conversion, confirming a self-accelerating process of CEA. The addition of Component C effectively modified the curing behavior, and CEA 8 with 30% Component C reduced curing time by 60%, enabling traffic reopening within half a day. The curing times were accurately predicted for each type of CEA using curing kinetic models based on autocatalytic and iso-conversional approaches. These findings will provide theoretical and practical guidance for high-performance airport runway overlays, supporting rapid repair, extended service life, and environmental sustainability.

## 1. Introduction

In 2024, the total passenger throughput at Chinese civil airports reached 1.459 billion, marking a 15.9% increase compared to 2023. Under the repeated impact of aircraft take-offs and landings, aggressive freeze–thaw cycles, and exposure to chemical deicers, cement concrete pavements gradually deteriorate and lead to cracking, spalling, and aggregate loss. As critical infrastructure, airport runways need to maintain high structural integrity and durability to ensure operational safety and efficiency, which has created an urgent demand for rapid and durable maintenance strategies for runway pavements, especially under the operational constraints of active airports [1].

To extend pavement life and restore functional integrity, asphalt overlays have become a widely adopted rehabilitation technique in airport runway maintenance [2]. Hot-mix asphalt (HMA), such as conventional SMA-13 mixtures, is often used due to its ease of construction and relatively short curing times [3]. However, HMA overlays have shown insufficient durability under the severe operational conditions of modern airports, especially under high aircraft loads and wide ambient temperature fluctuations [4,5]. Common distresses including reflective cracking and rutting indicate that traditional materials struggle to meet the long-term durability requirements of high-grade airport infrastructure.

To address these limitations, thermosetting asphalt binders, particularly epoxy asphalt and polyurethane-based composites, have attracted growing research attention [6,7,8]. These thermosetting binders form a rigid three-dimensional network through chemical curing, offering superior stiffness, fatigue resistance, and dimensional stability [9,10]. Epoxy asphalt, in particular, has demonstrated excellent adhesion, thermal stability, and cracking resistance [11,12,13]. The epoxy asphalt mixtures exhibit markedly superior fatigue life and rutting resistance, which can be several times higher than conventional HMA [14]. These characteristics make such mixtures an excellent candidate for long-life pavements exposed to repeated loading, especially in demanding applications such as steel bridge decks and high-stress airfield overlays [15,16,17]. However, conventional hot-cured epoxy asphalt requires high processing temperatures and long curing times [18], which pose challenges for large-scale application in airport maintenance, especially where continuous airport operations restrict repair windows.

Given the demand for “non-disruptive” airport runway maintenance, cold-mix thermosetting asphalt offers a promising solution [19]. These systems typically consist of liquid thermoset resins, curing agents, compatibilizers, and functional fillers and can be mixed and applied at ambient temperatures [20,21]. The cold-mix epoxy asphalt (CEA) significantly reduces construction energy demand and carbon emissions, supports fast strength development, and simplifies field operations [22,23]. Compared to hot-mix epoxy asphalt, CEA also offers improved storage stability and extended workability. Moreover, it allows for rapid reopening to traffic, which is critical in airport maintenance [24,25]. Previous studies have demonstrated that cold-mix asphalt shows satisfactory performance in both laboratory and field applications [26,27,28]. The compatibility of CEA with existing concrete runways can be considered from the following perspectives: mechanical properties, reflective cracking resistance, moisture stability, and interface bonding. The mechanical properties can be evaluated by tensile tests to obtain the basic strength and toughness. The reflective cracking resistance is the most important consideration for CEA overlays on cement concrete runways compared to asphalt runways, which can be evaluated by overlay tests (OTs). Although most studies focused on material properties, the moisture stability of CEA has been evaluated using residual stability (RS) and tensile strength ratio (TSR), both of which exceeded 90%, indicating strong resistance to moisture damage. As for the interface bonding, the epoxy bonding layers are generally used as bonding materials at the interface between CEA overlays and cement concrete runways, which have been proven to provide excellent adhesive strength, typically 3–5 times higher than conventional emulsified asphalt bonding layers [14]. Therefore, the CEA overlay is expected to provide a promising solution with excellent durability, simplified workability, and low-carbon benefits.

Nevertheless, these cold-mix materials are prone to brittleness at low temperatures, raising concerns about cracking resistance in overlays subject to thermal stress from diurnal and seasonal temperature variations [29,30,31]. They tend to exhibit poor toughness and low cracking resistance, and their high reactivity often sacrifices ductility for curing speed [32]. As overlays placed directly over cement concrete joints, stress concentration can cause premature reflective cracking, which will expand rapidly in a rigid CEA overlay and greatly affect its service life [33,34]. Moreover, the curing behavior of CEA plays a critical role in determining its applicability for airport overlays, as it directly governs the allowable time window for reopening to traffic. However, existing studies have largely focused on empirical viscosity evolution trends, with limited exploration into the fundamental curing mechanisms. As a result, there remains a significant gap in accurately predicting traffic opening times and providing theoretical support for construction scheduling. Therefore, optimizing the toughness and curing behaviors of cold-mix epoxy asphalt is essential for their successful application in airport overlays.

This study aims to identify a suitable cold-mix epoxy asphalt with high toughness for overlay applications in airport runways under non-disruptive construction. Eight commercially available cold-mix epoxy asphalt formulations were first evaluated by tensile tests using strength and elongation. Subsequently, overlay tests (OTs) were conducted to assess their reflective cracking resistance. Based on the results of tensile and overlay tests, two high-performing candidates (CEA 1 and CEA 8) were selected to investigate the curing behaviors using differential scanning calorimetry (DSC) tests under varying content of Component C as toughening and curing modifiers. Based on non-isothermal curing kinetics, reaction mechanisms and activation energies were analyzed, and predictive models of curing time were established. The results provide scientific and technical guidance for the development of cold-mix epoxy asphalt overlays with high toughness, short curing windows, and excellent durability, which will support safe and efficient airport runway maintenance.

## 2. Materials and Methods

### 2.1. Cold-Mix Epoxy Asphalt (CEA)

The cold-mix epoxy asphalt (CEA) used in this study was prepared by mixing three primary components: Component A (epoxy resin, polyurethane prepolymer, polyol, and compatibilizer), Component B (asphalt, curing agent, curing accelerator, compatibilizer, and stabilizer), and Component C (rheological modifiers, structural regulators, and curing rate controllers; optional depending on requirements). The primary resins of Component A were Bisphenol-A and Bisphenol-F epoxy resins, polyurethane prepolymers, and polyol esters. Cold-mix curing agents (Component B) included polyamides, aliphatic amines, cycloaliphatic amines, phenolic amines, and other modified amines. The asphalt binder used in this study was a pen 60/80 base asphalt.

In this study, eight distinct CEAs were selected, all provided by the manufacturer and designated as A1–A8 for Component A and B1–B8 for Component B. Each formulation was prepared following a standardized procedure to ensure consistency. The key properties of Components A1–B8 and Components B1–B8 are presented in Table 1 and Table 2, respectively.

**Figure 1:**
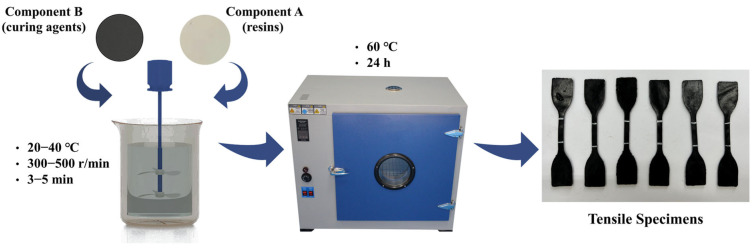
Preparation procedure for CEA.

To prepare the CEA, Components A and B were mixed at a mass ratio of 1:1.3 and sheared at 300 rpm for 1 min at 25 °C. The uncured CEA was then poured into molds and conditioned at 60 °C for 24 h for curing. The detailed preparation procedure is illustrated in Figure 1. Based on the types of Components A and B, the eight types of CEA were designated as CEA 1 to CEA 8.

### 2.2. Cold-Mix Epoxy Asphalt Mixture (CEAM)

For all eight combinations of Component A and Component B, cold-mix epoxy asphalt mixtures (CEAMs) were prepared based on the SMA-13 gradation, denoted as CEAM 1 to CEAM 8. The Marshall method was employed to determine the volumetric properties and the optimum epoxy asphalt content (OEAC). The immersion Marshall tests and freeze–thaw splitting tests were conducted to evaluate the moisture stability and temperature sensitivity of CEAM, where the residual Marshall stability (RS) and tensile strength ratio (TSR) were used as key performance indicators. The results are summarized in Table 3.

### 2.3. Experimental Methods

#### 2.3.1. Tensile Tests

The mechanical properties of eight cured CEAs were evaluated using tensile tests with a rate of 500 mm/min at 23 ± 1 °C based on ASTM D 638. Each sample was tested in six parallel replicates. The primary mechanical parameters were tensile strength ($T_{S}$) and elongation at break ($E_{b}$), determined using Equations (1) and (2), respectively.

(1) $$T_{S}=\frac{P}{B\times D}$$

where $T_{S}$ is the tensile strength, MPa; P is the maximum force, N; B is the middle width of the specimen, 6 mm; D is the thickness of the specimen, 2 mm.

(2) $$E_{b}=\frac{L_{1}-L_{0}}{L_{0}}\times 100$$

where $E_{b}$ is the elongation at break, %; $L_{0}$ is the original distance between the markers, 25 mm; $L_{1}$ is the distance between the markers at break, mm.

#### 2.3.2. Overlay Tests (OTs)

**Figure 2:**
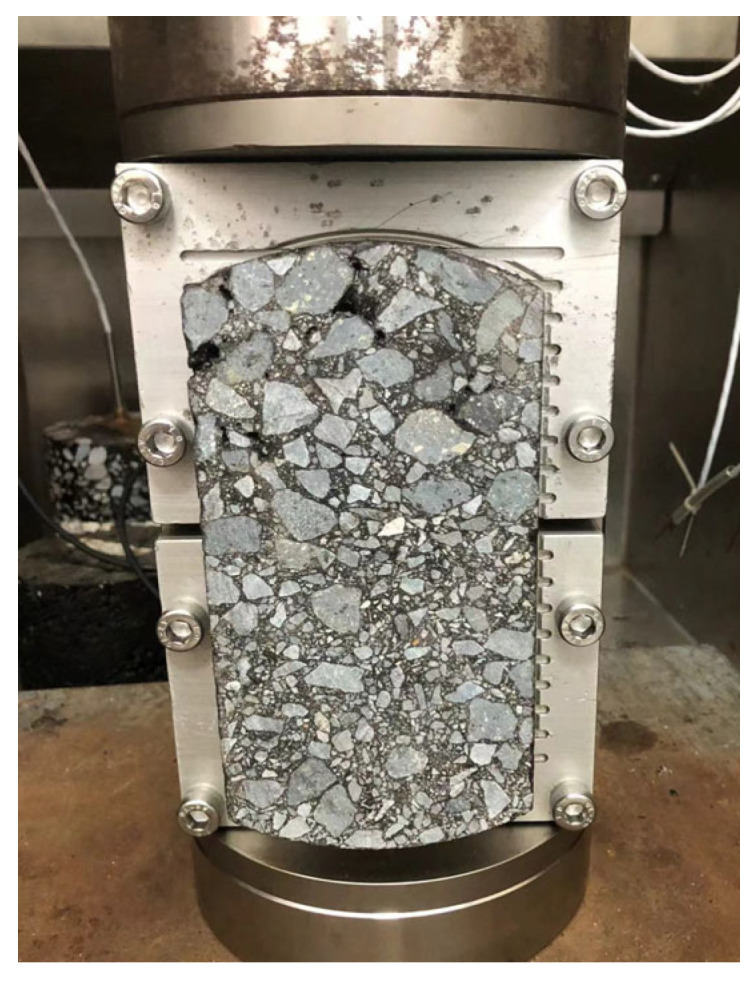
Specimen for overlay tests.

As an overlay on cement concrete runways, the reflective cracking resistance of asphalt mixtures is a critical factor. To assess this property, overlay tests (OTs) were performed on eight CEAMs across three temperatures (10 °C, 20 °C, and 30 °C). Test specimens were fabricated using a Superpave gyratory compactor (SGC) under a pressure of 600 kPa, a gyration angle of 1.25°, and 100 gyrations. The samples were cured at 60 °C for 24 h and then cut into specimens with 150 mm × 75 mm × 38 mm for the OTs, as shown in Figure 2. The OT induces Mode I crack in specimens subjected to tensile–compressive fatigue cycles with constant amplitude displacement (0.6 mm) and 0.1 Hz sinusoidal loading. The test was automatically terminated when the applied load drops to 7% of the initial load or when the load cycles reach 1000. The load cycles and the corresponding load reduction were recorded to evaluate the specimen’s resistance to reflective cracking.

#### 2.3.3. Differential Scanning Calorimetry (DSC) Tests

DSC tests were conducted using a DSC214 thermal analyzer (Netzsch, Bavaria, Germany) under a nitrogen atmosphere with a flow rate of 20 mL/min. The curing behaviors of CEA 1 and CEA 8 with different contents of Component C (0%, 10%, 20%, 30%), serving as toughening agents, were characterized. The uncured CEA samples were scanned from 0 °C to 200 °C at heating rates of 5, 10, 20, and 30 K/min, using aluminum crucibles as reference pans.

In this study, the curing temperatures were limited within 200 °C to reflect realistic processing and field application conditions for CEA. On the one hand, the mixing and curing temperatures of CEA for airport overlay are generally at ambient or slightly elevated temperatures (below 100 °C). On the other hand, temperatures above 200 °C will lead to thermal degradation of epoxy resins and curing agents. The temperatures below 200 °C can protect the DSC equipment from damage by decomposition products while ensuring complete exothermic conversion and accurate kinetic modeling.

## 3. Results and Discussion

### 3.1. Tensile Tests

**Figure 3:**
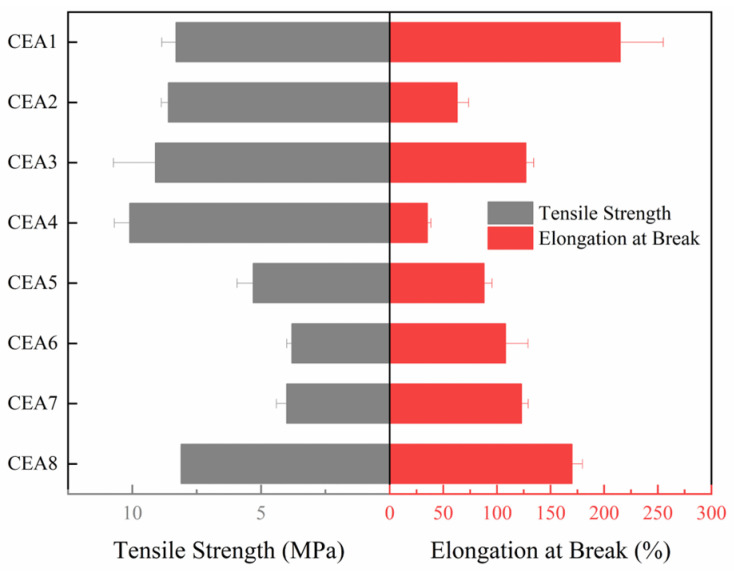
Tensile strengths and elongations at break of eight CEAs.

The tensile test is a basic test to evaluate the strength and toughness of epoxy asphalt. Generally, epoxy asphalt exhibits an obvious inverse relationship between tensile strength and elongation at break due to the rigid cross-linked network. As the tensile strength increases, the elongation at break tends to decrease. The tensile test results of eight CEAs are shown in Figure 3, differing significantly in strength and toughness.

Among the eight formulations, CEA 1, CEA 3, and CEA 8 demonstrate superior performance in both tensile strength and elongation at break, achieving 8.1 MPa/170% and 8.3 MPa/215%, respectively. These results suggest higher cross-linking efficiency and improved phase compatibility. The enhanced performance may be attributed to a better match between the molecular weight and functionality of the thermosetting resin components as well as the reactivity of the selected curing agents. These factors are likely to contribute to a more uniform cross-linked network structure, reducing the stress concentration within the polymer matrix. CEA 2 and CEA 4 exhibit high tensile strength but extremely low elongation at break, indicating a stiffer system with a dense cross-linked network. The restricted molecular mobility in these formulations limits their capacity for energy dissipation, resulting in limited toughness. In contrast, CEA 5, CEA 6, and CEA 7 exhibit approximately half the tensile strength but significantly higher elongation at break. This behavior is attributed to the formation of a more flexible network structure, primarily due to the use of low-rigidity curing agents. The increased chain mobility in these systems enhances ductility but compromises mechanical strength.

To clarify the individual roles of Components A, B, and C in influencing the mechanical and curing behaviors of cold-mix epoxy asphalt, their contributing factors to each test were analyzed. Component A, composed of epoxy resin, polyol, and polyurethane prepolymer, provides the main cross-linked network structure and affects the material type and cross-linking density of the final matrix. In this study, the Component A of eight CEAs has a similar composition, dominated by Bisphenol-A, and the CEAs mainly rely on Component B to regulate mechanical properties. Component B, mainly amine curing agent, controls the rigidity and toughness of CEA through the chain segments of the component matrix and regulates the cross-linking density through the ratio of reactive functional groups (A:B). For instance, CEA 4 shows high strength but brittle failure due to the use of highly rigid aliphatic amine in B4, while B8 allows more flexibility. It also determines the rate and extent of the thermoset reaction. Therefore, Component B plays the most critical role in the mechanical properties (tensile and overlay tests) of CEA. Component C, used as a rheological and curing modifier, influences the activation energy and curing time. The DSC analysis in Section 3.3 shows how increasing Component C content from 0% to 30% leads to changes in peak curing temperature, activation energy, DSC curve, and the curing time. Therefore, Component C plays a significant role in the curing behaviors (DSC tests) of CEAs.

**Figure 4:**
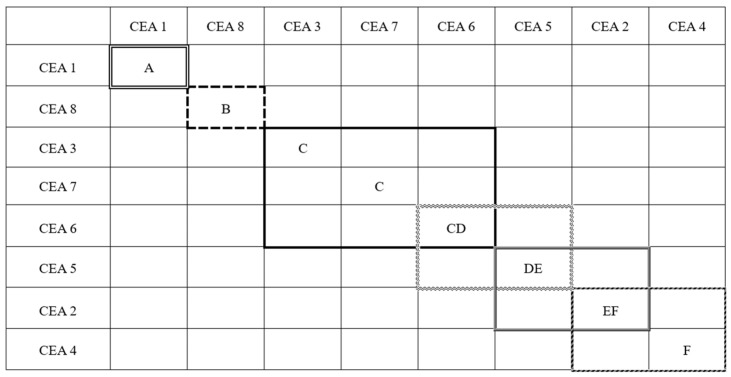
Tukey’s comparison for CEAs based on elongation at break.

According to “Specifications for epoxy asphalt pavement design and construction of airports” (MH/T5041-2019) [35], the tensile strength of cured epoxy asphalt at 23 °C should not be less than 1 MPa, and the elongation at break should not be less than 100%. Although all CEAs exhibit tensile strength values above the minimum requirement of 2 MPa, CEA 2, CEA 4, and CEA 5 with more rigid epoxy systems and higher cross-linking densities, fail to meet the requirement of elongation at break and demonstrate limited toughness. The brittle CEAs can significantly reduce the overlay’s ability to resist crack propagation when used over cement concrete pavements, especially in low-temperature regions. Therefore, the toughness is a critical parameter for selecting optimal materials for airport runway overlays. To quantitatively compare the performance among the eight CEAs and conduct material optimization, Tukey’s comparison was carried out to rank the elongation at break of the eight CEAs based on tensile test results. CEAs with elongations at break that were not significantly different between groups were categorized into the same level. The Tukey’s comparison results are shown in Figure 4, where ‘A’ represents the best toughness and ‘F’ stands for the worst. It shows that the CEAs were divided into six levels, and CEA 1 and CEA 8 exhibit the best toughness with a ranking of ‘A’ and ‘B’, respectively. Therefore, based on the principle of maximizing toughness, CEA 1 and CEA 8 are recommended as the most suitable formulations for runway overlay.

### 3.2. Overlay Tests

Cement concrete pavements at major airports are subjected to heavy single-wheel loads exceeding 260 kN and experience wide temperature gradients from −30 °C to 45 °C. Under daily thermal cycling, the transverse and longitudinal joints undergo cyclic displacements of approximately 0.5 mm. Combined with aircraft landing impacts of 3~5 Hz, these conditions typically lead to reflective cracking in asphalt overlays within just 2~5 years. Reflective cracking originates from the cement concrete base and propagates upward through the asphalt overlay. Its formation can be attributed to horizontal joint movement caused by thermal fluctuations and bending or shear stress induced by dynamic aircraft loads. In asphalt overlay applications for airport pavements, significant displacements occur at cement concrete joints due to the combined effects of diurnal temperature variation and repeated aircraft loading. These displacements create stress concentrations at the interface between asphalt overlay and cement base, initiating microcracks that progressively propagate to the surface as energy accumulates at the crack tip.

**Figure 5:**
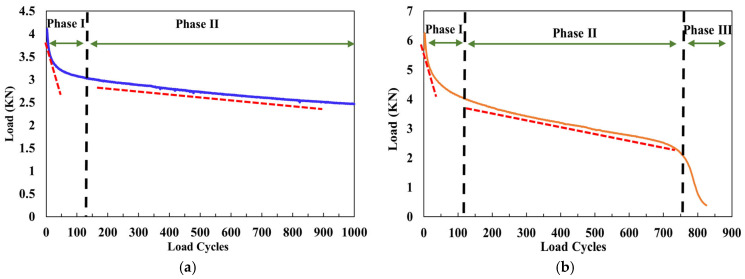
Typical load cycle curves in overlay testing: (**a**) two-phase damage; (**b**) three-phase damage.

Therefore, the reflective cracking resistance of cold-mix epoxy asphalt mixtures (CEAMs) is a critical indicator for use in airport overlays, which reflects the ability to inhibit crack initiation, delay crack propagation, and dissipate fracture energy. In this study, the reflective cracking resistances of eight CEAMs were evaluated using overlay tests (OTs) under three representative temperatures (10 °C, 20 °C, and 30 °C). Typical load cycle curves in OT are shown in Figure 5, including two-phase damage and three-phase damage. Phase I is characterized by a rapid load drop, indicating crack initiation at the notch tip and the localized plastic zone. Phase II represents the stable crack propagation stage, generally exhibiting linear decline in the load curve where the material maintains a relatively consistent damage accumulation rate. For those with less than 1000 load cycles, Phase III is clearly identifiable with a sudden drop, representing fracture instability with an inflection point between Phase II and Phase III. In contrast, CEAMs that sustain the full 1000 cycles remain within Phase II without entering the instability phase. The results of load cycles and load reduction in overlay testing are summarized in Table 4. A higher load cycle or a lower load reduction indicates improved resistance to reflective cracking.

With respect to the effects of temperature, the CEAMs exhibit a significant increase in fatigue life and a notable decrease in load reduction as the test temperature increases. This trend can be attributed to the viscoelastic nature of epoxy asphalt. At lower temperatures, the CEAM becomes stiffer and more brittle due to reduced molecular mobility, which limits its ability to dissipate energy. As a result, stress concentrations induced by joint opening displacements cannot be effectively relieved, making it more susceptible to cracking initiation and propagation. It is worth noting that the deterioration of the load reduction with decreasing temperature is not linear for some CEAMs such as CEAM 2 and CEAM 7, which decrease sharply from 20 °C to 10 °C, which implies that the glass transition temperature (Tg) exists in this temperature interval.

However, CEA 4, CEA 6, and CEA 2 fail prematurely at 251, 546, and 927 cycles, respectively, with high load reductions exceeding 90%, suggesting poor energy dissipation and brittle fracture behavior. This is consistent with the tensile test results that CEAs with low elongations at break are less able to resist reflective cracking. Although performance improved at higher temperatures, these formulations remain below optimal toughness due to excessive brittleness under cold conditions. These results reflect the inherent limitation of highly cross-linked or rigid networks at low temperatures, where molecular mobility is suppressed, resulting in minimal stress relaxation and rapid crack propagation.

As the temperature increases, a clear improvement in reflective cracking resistance was observed. At 20 °C, all CEAMs except CEA 4 reach 1000 cycles, and load reductions decrease significantly, reflecting improved viscoelastic properties. At 30 °C, thermal softening further enhances cracking resistance, with all CEAMs completing 1000 cycles. CEA 8 showed exceptional performance with a minimal load reduction of just 3.0%, followed by CEA 3 (15.1%) and CEA 1 (20.4%). The almost constant load of CEA 8 suggests a continuous and flexible cross-linked network as temperature increases, contributing to enhanced stress dissipation and cracking resistance. CEA 3 also sustains 1000 cycles across all temperatures with an average load reduction of 21.1%, though its 30 °C performance was inferior to CEA 8. CEA 1, which performs well in the tensile tests, exhibits moderate but smooth temperature-dependent toughness. While not as ductile as CEA 8 or CEA 3, its balanced response across the full temperature spectrum suggests good applicability in regions with large temperature variations. In line with the lowest elongation at break, CEA 4 is the only formulation that fails before 300 cycles at 10 °C, with consistently high load reductions ≥ 84% across all temperatures. It indicates that an excessively rigid cross-linked matrix, which restricts energy absorption and strain accommodation, will result in poor resistance to reflective cracking and is, therefore, unsuitable for airport runway overlay.

Based on fatigue life and load retention, the CEAMs were manually categorized as Grade A for CEA8 and CEA3, Grade B for CEA1, Grade C for CEA5, and Grade D for CEA 7, CEA 2, CEA 6, and CEA 4. From an engineering perspective, CEA 8 demonstrates strong potential for applications requiring high cracking resistance under wide temperature ranges and heavy traffic loads, such as stress-absorbing membrane interlayers (SAMI) or overlays on rigid airport runways. Its ability to retain structural integrity and prevent reflective cracks makes it especially suited for airfield pavements with frequent landings. CEA 1, while slightly more temperature sensitive, remains a reliable option for northern climates or regions with large day–night temperature fluctuations. For Grades B and C, performance may be improved by incorporating crumb rubber, mineral fillers, or other toughening agents to enhance low-temperature flexibility and energy dissipation. Additionally, optimized material design with chemical agents may reduce stress concentrations and delay microcrack initiation. For those belonging to Grade D, structural improvements should begin with adjusting the material matrix. A flexible network and an appropriate A:B ratio should be constructed to increase toughness and prevent premature cracking.

Based on the results of the tensile tests and overlay tests, CEA 1 and CEA 8 were identified as the most promising formulations for airport runway overlay applications. To further understand their performance and ensure construction reliability, the curing behaviors and reaction kinetics were investigated in the subsequent sections.

### 3.3. Curing Behaviors Based on DSC Tests

In field applications of CEAM overlays for airport runways, curing behaviors directly influence both construction workable time and long-term performance. It is critical for CEAMs to balance the need for rapid strength development with sufficiently wide construction time. When curing proceeds too rapidly, viscosity increases prematurely, limiting the workable time and compromising pavement quality. Conversely, if the cure is too slow, pavements will suffer from a late opening time to traffic, especially for time-sensitive maintenance in high-traffic airports.

Based on the tensile and overlay test results, CEA 1 and CEA 8 were selected for further investigation of their curing kinetics. To further enhance their mechanical properties and suitability for concrete pavement overlays, a toughening modifier (Component C) was added with different proportions (0%, 10%, 20%, and 30%), by weight of Component A. The corresponding materials were denoted as CEA 1-0/10/20/30%, and CEA 8-1-0/10/20/30%. In this paper, DSC tests were performed to evaluate the curing process of those eight formulations of CEA 1 and CEA 8 under four non-isothermal heating rates (5, 10, 20, and 30 K/min). The reaction conversion (α) and peak temperature ($T_{P}$) can be obtained from heat flow curves. These experimental results were further fitted using classical non-isothermal kinetic models to quantify the curing dynamics. The autocatalytic model was applied to describe the reaction mechanisms, which has proven to be effective for epoxy resins. Additionally, key kinetic parameters such as activation energy ($E_{a}$) and the pre-exponential factor (A) were calculated using linearized forms of the Kissinger equation and the Ozawa method. This approach allows a robust comparison of the reactivity and curing behaviors of different CEAs and provides a predictive basis for optimizing curing schedules under variable field conditions.

The basic assumptions of the DSC-based kinetic modeling are as follows: (1) The measured heat flow from DSC testing is directly proportional to the rate of reaction progress, and the total heat released is uniquely related to the degree of conversion. (2) In terms of kinetic modeling, the double-parameter autocatalytic model (Sesták–Berggren equation) assumes that the reaction mechanism can be effectively described by a macroscopic rate function of the form $f(\alpha )=\alpha^{m}(1-\alpha {)}^{n}$, where α is the conversion degree. It further assumes that the reaction is chemically controlled throughout the curing process and does not transition into a diffusion-limited regime, even at high degrees of conversion. (3) Activation energy ($E_{a}$) is calculated using the iso-conversional Starink method, which assumes a constant $E_{a}$ within narrow α intervals. (4) The Málek method is employed to confirm the suitability of the autocatalytic model by analyzing the peak locations of characteristic functions z(α) and $y\left(\alpha \right)$. If these functions satisfy the condition $\alpha_{M}<\alpha_{P}<\alpha_{P}^{\infty }$ with $\alpha_{P}^{\infty }\neq 0.632$, the curing reaction is well described by an autocatalytic mechanism. (5) The model does not account for potential deviations caused by vitrification effects, localized phase separation, network inhomogeneities, or physical cross-linking.

**Figure 6:**
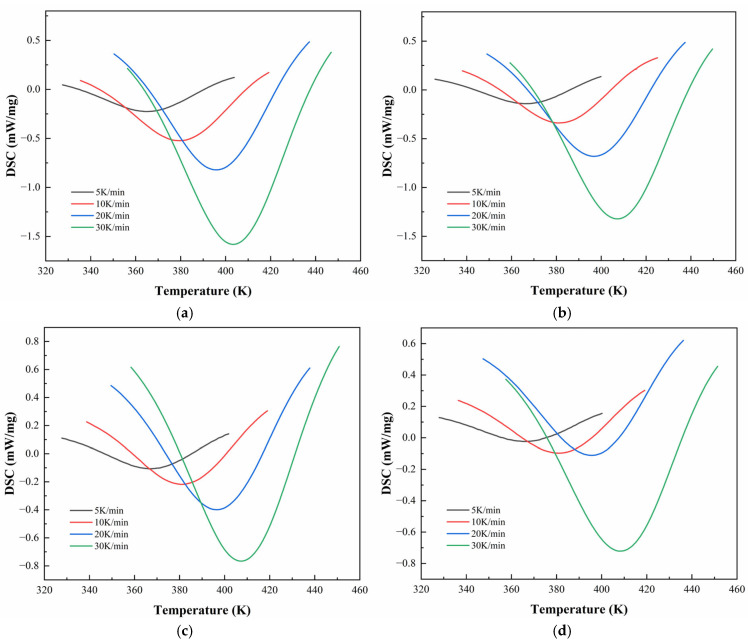
DSC test curves for CEA 1 with Component C contents of (**a**) 0%; (**b**) 10%; (**c**) 20%; (**d**) 30%.

**Figure 7:**
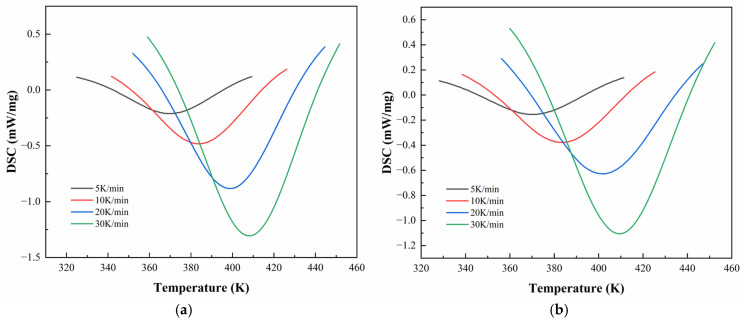

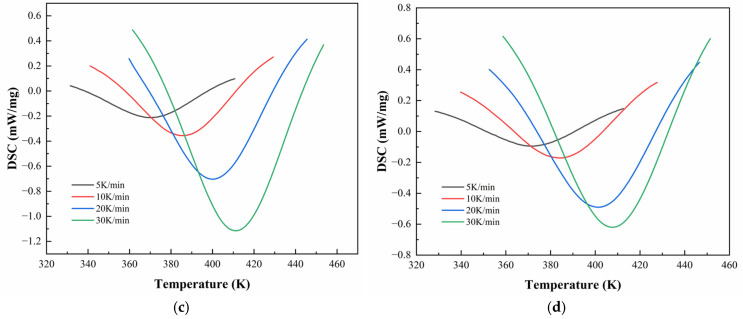
DSC test curves for CEA 8 with Component C contents of (**a**) 0%; (**b**) 10%; (**c**) 20%; (**d**) 30%.

Figure 6 and Figure 7 illustrate the DSC thermograms of CEA 1 and CEA 8 incorporating different proportions of component C under four heating rates. All curves exhibit a single exothermic peak, indicating that both materials follow a dominant one-step curing reaction, consistent with an autocatalytic mechanism. The peak temperature ($T_{P}$) systematically shifts toward higher temperatures with increasing heating rates, reflecting the kinetic delay associated with thermal diffusion and reaction initiation.

The detailed temperatures corresponding to DSC peaks ($T_{P}$) are summarized in Table 5. For CEA 1, $T_{P}$ increases from 364.6 K to 403.1 K across heating rates, while CEA 8 exhibits slightly higher $T_{P}$ values ranging from 369.4 K to 411.5 K. Among various formulations, the incorporation of component C (a reactive toughening agent) leads to a rightward shift in $T_{P}$ for both materials, accompanied by a clear reduction in peak height and narrowing of the exothermic curve. This suggests that component C may also serve as a kinetic modulator, which helps moderate the curing rate at moderate levels (10–20%), extending the processing window without sacrificing thermal reactivity. In addition, CEA 1 without Component C demonstrates a lower initial $T_{P}$ and higher exothermic enthalpy than CEA 8, indicating a faster cure initiation and higher early-stage cross-linking rate. However, after the addition of Component C, the difference in $T_{P}$ between CEA 1 and CEA 8 narrows, effectively aligning their curing windows and improving processing control.

The non-isothermal curing kinetics of the selected CEA 1 and CEA 8 with varying Component C content were quantitatively evaluated using DSC data, based on the assumption that the rate of the epoxy curing reaction is proportional to the heat flow rate. The fundamental kinetic model is given by Equation (3):

(3) $$\frac{d\alpha }{dt}=K\left(T\right)f\left(\alpha \right)$$

where α is the curing degree or conversion rate, t is time, s, $\frac{d\alpha }{dt}$ is the curing reaction rate, $K\left(T\right)$ is the temperature-dependent reaction rate constant, and $f\left(\alpha \right)$ is the kinetic model function. The temperature dependency of the rate constant $K\left(T\right)$ follows the Arrhenius equation:

(4) $$K(T)=Aexp(-\frac{E_{\alpha }}{RT})$$

where A is the pre-exponential factor (s^−1^), $E_{\alpha }$ is the apparent activation energy (J/mol), R is the universal gas constant (8.314 J/mol·K), and T is absolute temperature (K).

Combining both relationships yields the general form of the epoxy curing process:

(5) $$\frac{d\alpha }{dt}=Aexp\left(-\frac{E_{a}}{RT}\right)f\left(\alpha \right)$$

The apparent activation energy $E_{a}$ is a key kinetic parameter that reflects the difficulty of the curing process. A lower $E_{a}$ indicates easier reaction progression, while a higher $E_{a}$ corresponds to a more sluggish reaction. Since $E_{a}$ is not constant during the entire curing process, its evolution with the curing degree (α) can be studied using the iso-conversional method. The Starink method is commonly employed for this purpose and is expressed as [32]

(6) $$\ln\left(\frac{\beta }{T_{\alpha }^{1.92}}\right)=C-1.0008\frac{E_{a}}{RT_{\alpha }}$$

where β is the heating rate, $T_{\alpha }$ is the peak temperature corresponding to a fixed conversion α, and $E_{a}$ is the activation energy at that conversion. By plotting $\ln\left(\frac{\beta }{T_{\alpha }^{1.92}}\right)$ against $\frac{1}{T_{\alpha }}$ for multiple heating rates, the slope of the linear fit allows direct calculation of $E_{a}$ at different curing stages.

**Figure 8:**
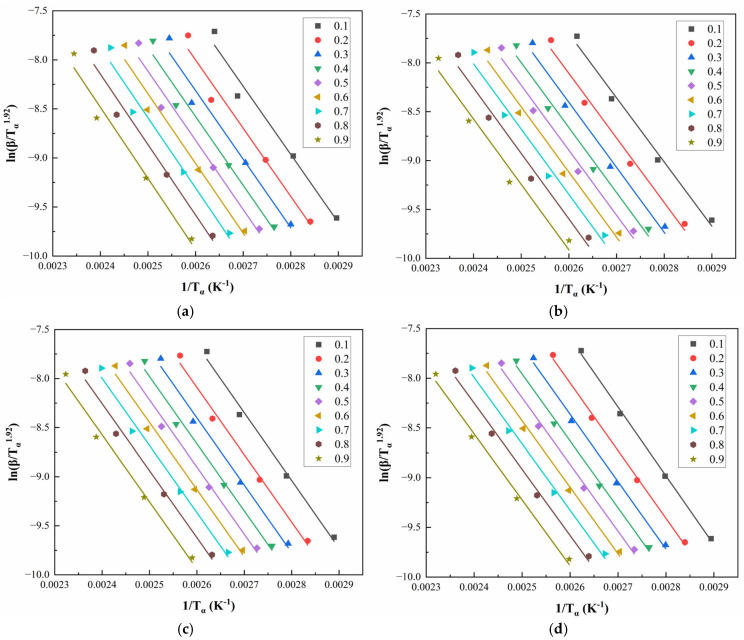
Starink method for solving $E_{\alpha }$ of CEA 1 with Component C contents of (**a**) 0%; (**b**) 10%; (**c**) 20%; (**d**) 30%.

**Figure 9:**
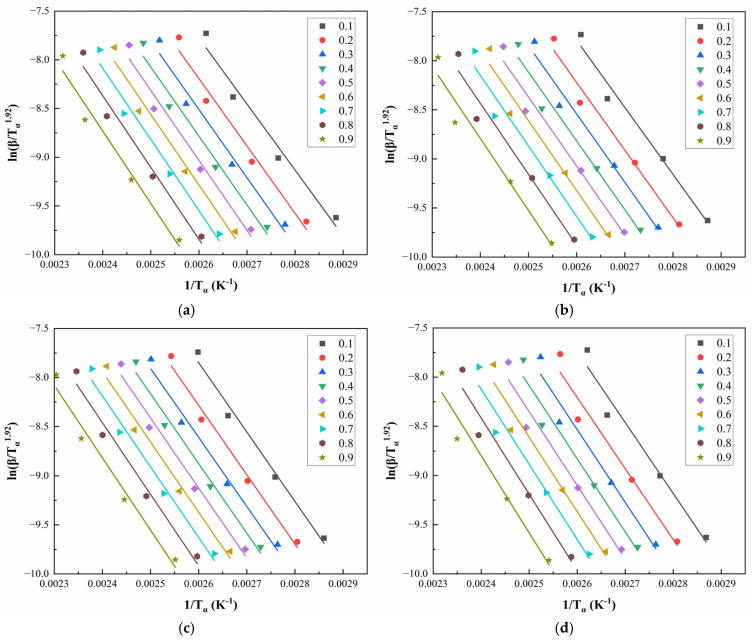
Starink method for solving $E_{\alpha }$ of CEA 8 with Component C contents of (**a**) 0%; (**b**) 10%; (**c**) 20%; (**d**) 30%.

Figure 8 and Figure 9 illustrate the linear regression results using the Starink method for solving $E_{\alpha }$ for CEA 1 and CEA 8, respectively. The calculated activation energy values exhibit a decreasing trend with increasing α, suggesting that the curing reaction becomes progressively easier as it proceeds. This phenomenon may be attributed to two coupled effects. On the one hand, viscosity reduction during early-stage heating improves molecular mobility, thus lowering the energy barrier for molecular diffusion. On the other hand, hydroxyl groups generated during the initial reaction stages may act as catalysts, further reducing the overall activation energy required for continuation. This confirms the autocatalytic nature of the CEA curing process and provides theoretical support for optimizing heating schedules in practical applications.

To describe the complex curing behavior of CEA systems, the selection of an appropriate reaction model is crucial. Previous studies have demonstrated that the curing of thermosetting resins such as epoxy resins is well characterized by an autocatalytic model with two kinetic parameters. In this study, the double-parameter autocatalytic model was adopted as the reaction function $f\left(\alpha \right)$, as shown in Equation (7).

(7) $$f(\alpha )=\alpha^{m}(1-\alpha {)}^{n}$$

Substituting into the general rate equation leads to the full kinetic model:

(8) $$\frac{d\alpha }{dt}=Aexp(-\frac{E_{a}}{RT})\alpha^{m}{\left(1-\alpha \right)}^{n}$$

**Figure 10:**
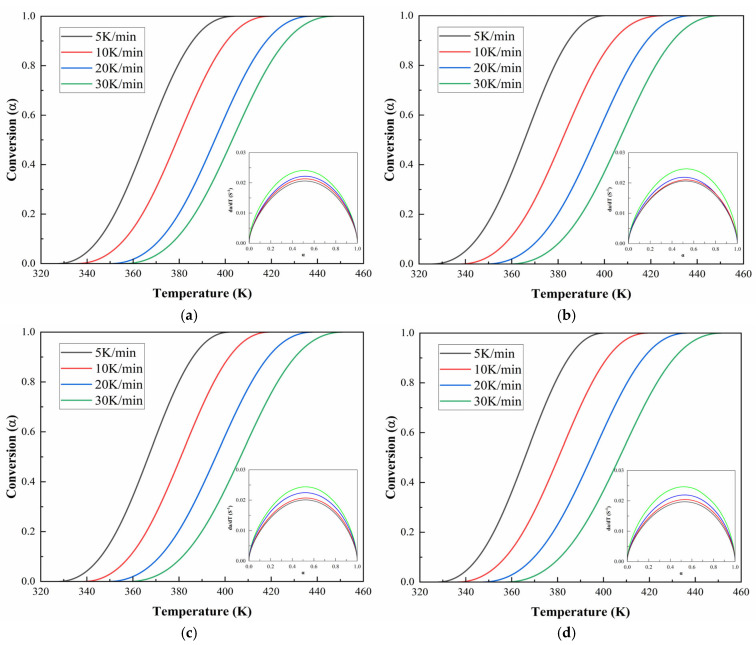
Conversion temperature curves of CEA 1 with Component C contents of (**a**) 0%; (**b**) 10%; (**c**) 20%; (**d**) 30%.

**Figure 11:**
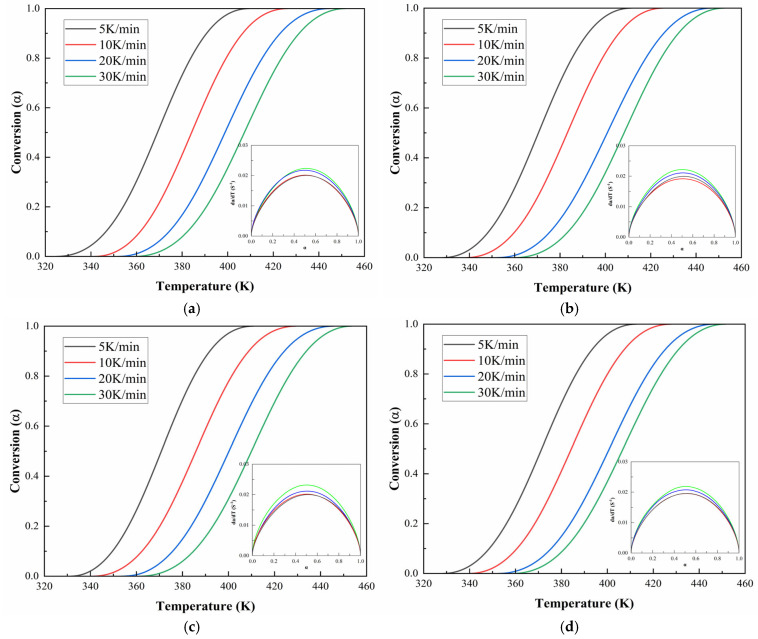
Conversion temperature curves of CEA 8 with Component C contents of (**a**) 0%; (**b**) 10%; (**c**) 20%; (**d**) 30%.

Figure 10 and Figure 11 demonstrate the evolution of curing degree (α) with temperature under various heating rates for CEA 1 and CEA 8, respectively. The results reveal that at any given temperature, higher heating rates lead to lower curing degrees and sharper conversion curves. This indicates that increasing the heating rate not only accelerates the curing process but also compresses the reaction window, reducing the available working time. The α-T profiles for both CEA 1 and CEA 8 show typical S-shaped autocatalytic reactions. Initially, the conversion remains low, marking the induction period corresponding to early-stage gelation. As the temperature rises, the reaction accelerates rapidly due to increased molecular mobility and greater accessibility of reactive groups, facilitating the formation of a cross-linked three-dimensional network. This middle stage is also where vitrification may begin to occur. As the system approaches the final phase, the depletion of reactive functional groups and the rise in viscosity limit further reaction progress, causing the curing rate to slow and plateau.

This behavior is further supported by the reaction rate curves, where $\frac{d\alpha }{dt}$ is plotted against α. These curves exhibit a bell-shaped profile, with the curing rate peaking at around α = 0.5. This indicates that the maximum rate of network formation occurs near the midpoint of the reaction, after which the declining availability of reactants cause a gradual drop in reactivity. The figures strongly support the adoption of a double-parameter autocatalytic model to describe the system kinetics. Moreover, the temperature dependence of the curing rate must be carefully managed in practical applications: although elevated temperatures accelerate curing, Van’t Hoff’s law suggests that the equilibrium conversion of an exothermic reaction may decrease as temperature increases. Therefore, optimization requires balancing between rapid early-stage reaction and high final conversion, particularly in time-sensitive construction environments.

To rigorously determine the reaction model and extract kinetic parameters, the Málek method was applied [33], which helps to determine two key parameters:

(9) $$y\left(\alpha \right)=\left(\frac{d\alpha }{dt}\right)\exp\left(x\right)$$

(10) $$z(\alpha )=\pi (x)(\frac{d\alpha }{dt})(\frac{T}{\beta })$$

where $x=\frac{E_{a}}{RT}$, and π(x) is an integral form of the temperature, calculated by Equation (11),

(11) $$\pi (x)=\frac{x^{3}+18x^{2}+88x+96}{x^{4}+20x^{3}+120x^{2}+240x+120}$$

According to the criteria proposed by Málek, the corresponding conversion degrees at their respective peak values of $y\left(\alpha \right)$, z(α), and $\frac{d\alpha }{dt}$ are denoted as $\alpha_{M}$, $\alpha_{P}^{\infty }$, and $\alpha_{P}$. If these functions satisfy the condition $\alpha_{M}<\alpha_{P}<\alpha_{P}^{\infty }$ with $\alpha_{P}^{\infty }\neq 0.632$, the curing reaction is well described by an autocatalytic mechanism. This condition was met for both the CEA 1 and CEA 8 systems under all tested conditions, further confirming the applicability of the autocatalytic model for these cold-mix epoxy asphalts.

To determine the kinetic parameters, Equation (8) was logarithmically transformed to allow linear regression fitting:

(12) $$\ln\left[\left(\frac{d\alpha }{dt}\right)\exp\left(-\frac{E_{a}}{RT}\right)\right]=lnA+nln\left[\alpha^{p}\left(1-\alpha \right)\right]$$

In this expression, the apparent activation energy $E_{a}$ can be obtained from Starink analysis, and the term $p=\frac{m}{n}=\frac{\alpha_{M}}{1-\alpha_{M}}$ was introduced to simplify the regression. Using data within the conversion range α∈(0.2, 0.8), $\ln\left[\left(\frac{d\alpha }{dt}\right)\exp\left(-\frac{E_{a}}{RT}\right)\right]$ was plotted against $ln\left[\alpha^{p}\left(1-\alpha \right)\right]$, and linear fitting was performed. The slope of the resulting line provided the overall reaction order n, from which the secondary exponent m can be calculated. The pre-exponential factor A was derived from the intercept.

The resulting parameters for each CEA (reaction exponents m, n, and pre-exponential factor A) are summarized in Table 6. These values were then substituted into the kinetic expression to establish the complete curing model equations for each CEA formulation, as shown in Table 7. CEA 8 exhibits slightly higher apparent activation energy ($E_{a}$) than CEA 1, suggesting a more energy-demanding network initiation in CEA 8 systems. Component C has different effects on the $E_{a}$ values of CEA 1 and 8, perhaps due to different toughening reactions caused by different resin matrixes. For CEA 1, the addition of Component C results in a lower $E_{a}$, while the highest $E_{a}$ (62.2 kJ/mol) was observed in CEA 8 with 30% Component C, indicating that it notably impedes the molecular mobility and slows down the early-stage reaction of CEA 8.

The $\alpha_{M}$ and $\alpha_{P}^{\infty }$ values from the Málek method further support the autocatalytic nature of the curing process, as all CEAs satisfy the criteria $\alpha_{M}<\alpha_{P}<\alpha_{P}^{\infty }$ with $\alpha_{P}^{\infty }\neq 0.632$, confirming that the dual-parameter autocatalytic model is well suited for describing the complex cross-linking behaviors in this study. The reaction orders m and n show that most systems maintain consistent autocatalytic features, with m ranging from 0.157 to 0.207 and n ranging from 1.116 to 1.288. The relatively high n values indicate that diffusion-controlled effects and network growth dominate the later stages of curing. Compared with CEA 1, the CEA 8 series tends to exhibit larger n values and lower m values, especially at higher Component C contents, suggesting a more constrained cross-linking mechanism with slower nucleation but faster propagation once activated. In practice, these distinctions imply that CEA 1–20% and CEA 8–20% offer balanced reactivity and control, making them ideal candidates for applications requiring moderate cure speed with sufficient working time. On the other hand, CEA 8–30% exhibits superior control but may require extended curing time or elevated curing temperatures to reach full cross-link density.

**Figure 12:**
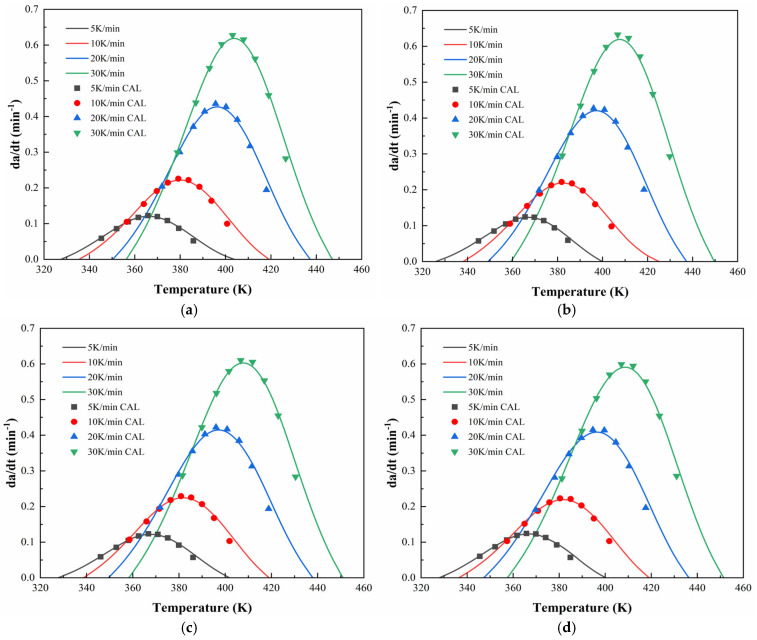
Calculated and experimental rates of CEA 1 with Component C contents of (**a**) 0%; (**b**) 10%; (**c**) 20%; (**d**) 30%.

**Figure 13:**
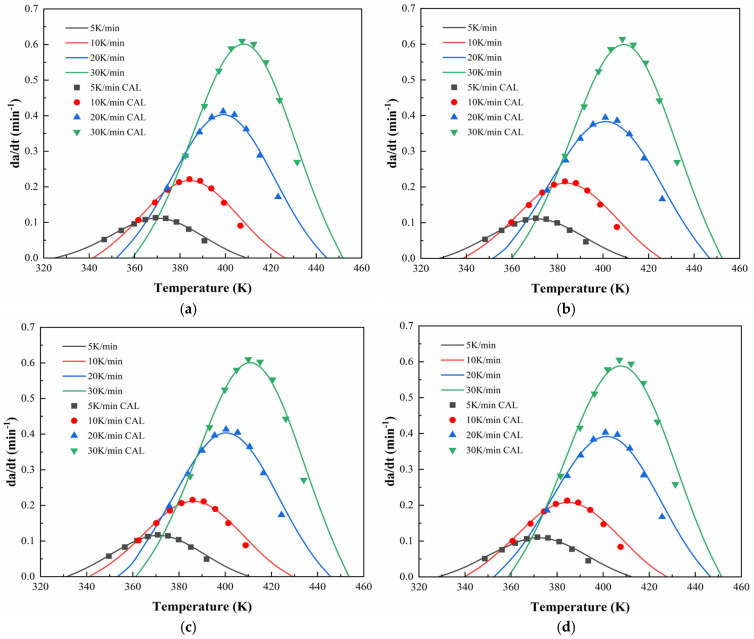
Calculated and experimental rates of CEA 8 with Component C contents of (**a**) 0%; (**b**) 10%; (**c**) 20%; (**d**) 30%.

To further validate the applicability of the proposed dual-parameter autocatalytic model, Figure 12 and Figure 13 compare the model-predicted curing rate curves with experimental data for CEA 1 and CEA 8, respectively. The close alignment between the calculated and experimental curves confirms the accuracy of the curing model in capturing the full curing behaviors under non-isothermal conditions. It indicates that the Sesták–Berggren equation, a generalized form of the autocatalytic model, provides a robust framework for describing the multi-stage curing behavior of cold-mix epoxy asphalt systems. Specifically, it accommodates both the acceleration phase dominated by autocatalytic effects and the deceleration stage constrained by diffusion and vitrification. As such, the validated kinetic formulation offers not only mechanistic insights but also practical guidance for curing schedules of CEAs to meet construction and performance demands.

Based on the established curing kinetic models for CEAs, the curing times required to reach a curing degree of 95% under different temperatures (50 °C, 60 °C, and 70 °C) were calculated and are summarized in Table 8. A clear temperature-dependent trend can be observed, where curing time decreases significantly with increasing temperature, consistent with Arrhenius kinetics. CEA 1 exhibits a shorter curing time at all temperatures compared to CEA 8, indicating a higher inherent reactivity and faster initiation of the curing process, which is consistent with previous parameter analyses. This suggests CEA 1 may be preferable for rapid maintenance applications where curing must proceed in a short time or at lower ambient temperatures.

The incorporation of Component C into both CEA 1 and CEA 8 results in marked reductions in curing time. For CEA 1, adding 10% Component C decreases the curing time at 50 °C by 26.2% from 24.8 h to 18.3 h; however, a further increase to 30% Component C does not continue to accelerate curing significantly for CEA 1. In contrast, CEA 8 responds more sensitively to the proportion of Component C. When the content is within 20%, there is no significant effect on curing times. However, a substantial shift occurs at 30% content, with curing time reduced by 60% across all curing temperatures. This can be attributed to the potential catalytic and network modifying roles of Component C, which may lower diffusion barriers or stabilize transitional structures during cross-linking. This demonstrates that despite a slower reaction initiation of CEA 8, the significant acceleration of Component C enables it to meet rapid-curing demands within half a day (only 4.1 h at 70 °C), confirming the role of Component C as a curing accelerator. This is of great importance for non-disruptive constructions of airport runway overlay where minimal closure time is critical.

Compared to conventional HMA or heat-cured epoxy systems, cold-mix epoxy asphalt has several economic and environmental advantages. (1) Cold-mix epoxy asphalt eliminates the need for high-temperature heating, thereby reducing energy consumption and greenhouse gas emissions during construction. (2) The selected CEA in this paper shows superior mechanical performance and durability, therefore enhancing the service life and reducing pavement maintenance, which will significantly decrease carbon emissions and energy consumption. (3) The shortened curing times enabled by Component C allow earlier reopening to traffic, minimizing operational disruption and associated indirect costs. These features collectively enhance the economic and environmental sustainability of CEA in airport overlay applications.

Overall, the kinetic models quantitatively characterize the curing behaviors under non-isothermal conditions and offer predictive capacity for practical applications such as open-time control, construction scheduling, and thermal management during field curing. The time estimation approach highlights the potential of CEA 1 and CEA 8 systems to meet varied application demands, from non-disruptive overlay construction to curing-controlled paving, by adjusting Component C content and curing temperature management.

## 4. Conclusions

This study systematically evaluated the mechanical performance, reflective cracking resistance, and curing kinetics of cold-mix epoxy asphalt (CEA) systems to identify optimal formulations for airport runway overlays and provide guidance for construction control. The key conclusions are as follows.

1. CEA 1 and CEA 8 were selected as optimal formulations, with both achieving tensile strengths > 7 MPa and elongations at break >170%, meeting the performance requirements for runway overlays.
2. Overlay tests results confirm that CEA 8 exhibits excellent resistance to reflective cracking across a wide temperature range, maintaining 1000 loading cycles with minimal load loss (<5% at 30 °C), attributed to its high glass transition temperature and stable viscoelastic network.
3. DSC-based curing kinetic analysis shows that the curing reactions of CEA follow a single-step autocatalytic mechanism, and activation energy decreases with conversion, confirming a self-accelerating process.
4. The developed curing kinetic models can accurately predict curing times for CEA under various temperatures, offering practical tools for construction scheduling in field airport overlay applications.
5. The addition of Component C effectively modifies the curing behaviors. For CEA 8, 30% Component C reduces the curing time by 60% and reaches 95% curing degree within 4 h at 70 °C, enabling traffic reopening within half a day and making it suitable for rapid, non-disruptive airport overlay construction.

## Figures and Tables

**Table 1:** Physical properties of Component A.

Component A	Viscosity (23 °C, mPa·s)	Appearance	Density (g/cm^3^)
A1	563	colorless transparent liquid	1.08
A2	697		1.16
A3	782		1.18
A4	906		1.09
A5	1082		1.02
A6	898		1.21
A7	1023		1.03
A8	657		1.06
Testing procedure	ASTMD4402	visually	ASTMD1475

**Table 2:** Physical properties of Component B.

Component A	Viscosity (23 °C, mPa·s)	Appearance	Density (g/cm^3^)
B1	437	black liquid	0.93
B2	398	black liquid	0.91
B3	622	black liquid	1.01
B4	712	dark brown liquid	0.97
B5	507	dark brown liquid	0.89
B6	489	black liquid	1.05
B7	531	black liquid	0.98
B8	512	black liquid	0.93
Testing procedure	ASTMD4402	visually	ASTMD1475

**Table 3:** Volumetric parameters and moisture test results of CEAMs.

Materials	OEAC (%)	Bulk Density (g/cm^3^)	Theoretical Maximum Density (g/cm^3^)	Air Voids (%)	Marshall Stability (kN)	Flow Value (0.1 mm)	RS (%)	TSR (%)
CEAM 1	6.0	2.499	2.582	3.2	47.08	29.3	92.3	92.5
CEAM 2	5.9	2.520	2.602	3.1	45.7	28.4	87.9	88.6
CEAM 3	6.0	2.501	2.579	3.0	97.6	28.9	93.7	94.5
CEAM 4	5.8	2.525	2.612	3.3	88.1	22.5	92.1	91.2
CEAM 5	5.7	2.522	2.604	3.2	85.0	26.3	89.2	88.7
CEAM 6	5.8	2.531	2.619	3.4	95.6	28.9	88.2	86.8
CEAM 7	5.8	2.513	2.587	3.0	35.1	30.1	91.1	90.3
CEAM 8	5.7	2.530	2.616	3.3	38.9	28.2	92	91.7

**Table 4:** Load cycles and load reduction of eight CEAMs in overlay tests.

Materials	10 °C		20 °C		30 °C	
	Load Cycles	Load Reduction	Load Cycles	Load Reduction	Load Cycles	Load Reduction
CEAM 1	1000	38.1% ± 3.0%	1000	29.9% ± 2.3%	1000	20.4% ± 1.9%
CEAM 2	927 ± 42	93.0%	1000	62.2% ± 4.1%	1000	53.6% ± 3.5%
CEAM 3	1000	29.9% ± 1.3%	1000	18.2% ± 1.7%	1000	15.1% ± 1.8%
CEAM 4	251 ± 49	93.0%	825 ± 53	93.0%	1000	84.0% ± 6.3%
CEAM 5	1000	46.7% ± 2.8%	1000	45.7% ± 2.2%	1000	38.1% ± 2.7%
CEAM 6	546 ± 35	93.0%	1000	77.2% ± 4.3%	1000	65.0% ± 3.4%
CEAM 7	1000	84.4% ± 4.5%	1000	54.2% ± 2.6%	1000	51.1% ± 2.9%
CEAM 8	1000	28.3% ± 3.1%	1000	13.4% ± 1.6%	1000	3.0% ± 0.8%

**Table 5:** Temperatures corresponding to DSC peaks ($T_{P}$) at different heating rates.

Materials	Heating Rate (K/min)			
	5	10	20	30
CEA 1–0%	364.62356	379.68934	395.81159	403.13056
CEA 1–10%	365.90808	380.68270	396.60749	407.32837
CEA 1–20%	366.62159	381.16321	396.41105	407.33544
CEA 1–30%	365.02263	381.08161	395.81289	407.92829
CEA 8–0%	369.4096	383.67868	399.38298	408.23234
CEA 8–10%	369.37396	383.58186	402.20572	409.43032
CEA 8–20%	369.72107	385.56587	400.38061	411.52359
CEA 8–30%	370.86162	383.87074	400.78427	407.33063

**Table 6:** Summary of fitted parameters for different CEA formulations.

Materials	$E_{a}$ (J/mol)	$\alpha_{M}$	$\alpha_{P}^{\infty }$	A	m	n
CEA 1–0%	58677	0.136	0.556	68895021	0.186	1.187
CEA 1–10%	55216	0.156	0.565	21044717	0.207	1.118
CEA 1–20%	56869	0.142	0.561	34767443	0.187	1.133
CEA 1–30%	55893	0.141	0.574	25772872	0.184	1.116
CEA 8–0%	59266	0.129	0.546	67495621	0.184	1.236
CEA 8–10%	58618	0.126	0.554	52220827	0.179	1.239
CEA 8–20%	59372	0.131	0.548	64119505	0.186	1.231
CEA 8–30%	62228	0.109	0.556	159280831	0.157	1.288

**Table 7:** Curing kinetic models for different CEA formulations.

Materials	Curing Kinetic Model
CEA 1–0%	$\frac{d\alpha }{dt}=6.89\times 10^{7}exp(-\frac{58677}{RT})\alpha^{0.186}{\left(1-\alpha \right)}^{1.187}$
CEA 1–10%	$\frac{d\alpha }{dt}=2.10\times 10^{7}exp(-\frac{55216}{RT})\alpha^{0.207}{\left(1-\alpha \right)}^{1.118}$
CEA 1–20%	$\frac{d\alpha }{dt}=3.47\times 10^{7}exp(-\frac{56869}{RT})\alpha^{0.187}{\left(1-\alpha \right)}^{1.133}$
CEA 1–30%	$\frac{d\alpha }{dt}=2.58\times 10^{7}exp(-\frac{55893}{RT})\alpha^{0.184}{\left(1-\alpha \right)}^{1.116}$
CEA 8–0%	$\frac{d\alpha }{dt}=6.75\times 10^{7}exp(-\frac{59266}{RT})\alpha^{0.184}{\left(1-\alpha \right)}^{1.236}$
CEA 8–10%	$\frac{d\alpha }{dt}=5.22\times 10^{7}exp(-\frac{58618}{RT})\alpha^{0.179}{\left(1-\alpha \right)}^{1.239}$
CEA 8–20%	$\frac{d\alpha }{dt}=6.41\times 10^{7}exp(-\frac{59372}{RT})\alpha^{0.186}{\left(1-\alpha \right)}^{1.231}$
CEA 8–30%	$\frac{d\alpha }{dt}=1.59\times 10^{8}exp(-\frac{58677}{RT})\alpha^{0.157}{\left(1-\alpha \right)}^{1.288}$

**Table 8:** Calculated curing times of CEA 1 and CEA 8 with different Component C contents.

Materials	Curing Times (h) at Different Curing Temperatures (°C)		
	50 °C	60 °C	70 °C
CEA 1–0%	24.8	12.9	7.0
CEA 1–10%	18.3	9.9	5.5
CEA 1–20%	21.4	11.3	6.2
CEA 1–30%	19.0	10.2	5.7
CEA 8–0%	36.5	18.8	10.1
CEA 8–10%	37.4	19.5	10.5
CEA 8–20%	39.4	20.3	10.9
CEA 8–30%	14.5	7.5	4.1

## Data Availability

The original contributions presented in this study are included in the article. Further inquiries can be directed to the corresponding author(s).
